# Miscellaneous Notices

**Published:** 1850-07-01

**Authors:** 


### Miscellaneous Notices.

*On the Causes, Symptoms, and Treatment of Eccentric Nervous Affections.* By William Anderson, F.R.C.S. London: Churchill. 1850.

We have been much pleased with this work. It is evidently the production of a reflecting, painstaking, and intelligent man. Dr. Marshall Hall has established that the diseases of the nervous system may be divided into those which have their origin at the nervous *centres,* and those which occur at a distance from these centres, in fact, into those *centric* and those *eccentric* in their character. Mr. Anderson proposes to confine himself to the latter class of affections, being, as he says, far more numerous than is generally supposed. The eccentric nervous affections are often formidable in their nature, and, alas! frequently intractable. They run their course with great rapidity, and give rise to the most frightfully violent symptoms. The introductory part of the work before us contains an admirable account of the circulating system, and an excellent resumé of the present state of our knowledge of the properties of the blood, of assimilation, and reflex action. He then proceeds to consider the pathological division of affections of the nervous system, and subsequently treats, at some length, the following diseases: *hysteria, epilepsy, delirium traumaticum, delirium tremens, hydrophobia,* &c.

We had marked several passages in Mr. Anderson's work for extract, but the crowded condition of our pages compels us reluctantly to deprive our readers of the pleasure and advantage they were sure to have derived from their perusal. We trust, however, the work itself will find its way into the hands of our friends. Mr. Anderson cites many cases illustrative of his peculiar views—many of these cases came under his observation during his pupilage at St. George's Hospital, where our author, we understand, studied his profession with great industry and zeal under the official instruction of that able physician, Dr. Seymour, who was at that period senior physician to the hospital.

*Health, Disease, and Remedy.* By George Moore, M.D., M.R.C.P. &c. London: Longman, Brown and Co. 1850.

In more than one number of this journal we have had occasion to speak most favourably of the writings of Dr. G. Moore, the well-known author of the work entitled, "The Power of the Soul over the Body."

The volume now under review, although in many of its features differing from the writer's previous productions, inasmuch as it refers less immediately to psychological subjects, is nevertheless one of great interest and value, particularly so at this time, when the great question of public health is under the consideration of the legislature. Dr. Moore writes scientifically, and, at the same time, intelligibly, on the art of preventing disease. The volume is replete with sound, judicious advice. It would be well for the community if the rules so clearly and forcibly laid down by the author, were literally followed. We are glad to see Dr. Moore run a tilt at homœopathy and its celebrated founder, the illustrious (!) Hahnemann, who had the genius to discover that the most formidable diseases have their origin in that "ancient miasm," the *itch!* Dr. Moore, however, qualifies his censures by saying that it is a consolation to think that "homœopathic medicines do not poison," and expresses his thanks to these practitioners "for their facts, as they (the homœopathists) teach us the importance of *doing nothing to thwart nature.*" We have first to learn that they are legitimately entitled to such thanks. *They* teach us not to "thwart nature!" The immortal Sydenham, the great Heberden, the illustrious Hunter, taught us this doctrine in unmistakable language long before Hahnemann dawned upon this poor benighted world, and dazzled us by the splendour of his pathological discovery! The evil of homœopathy, and, in fact, of every other description of quackery, consists mainly in the false confidence inspired to the exclusion of other and more efficacious and scientific remedies. A man with a serious disease allows his health to be trifled with under the vain delusion that a "*decillionth degree of the solution of nux vomica*" will restore him to health. As nothing is literally done to *cure* the disease or arrest its fatal progress, what was, perhaps, in the *first* stage, when under the care of the homœopathic practitioner, a *functional* disorder, and, consequently, easily removable by judicious means, becomes *organic,* and places the poor patient irretrievably beyond the reach of the most efficient treatment.

We regret to perceive that Dr. Moore is disposed to offer some qualified praise to another section of practitioners, the *hydropathists.* It is the bounden duty of all medical men who wish well to the community, and who are anxious to uphold the dignity and respectability of the profession, to discountenance in every way, and on all occasions, those who leave the beaten track and resort to empiricism, whatever garb it may assume.

*Elementary Sketches of Moral Philosophy, delivered at the Royal Institution, in the years* 1804-5-6. By the late Rev. SYDNEY SMITH, M.A. Longman. 1850.

IT was our intention to have laid before our readers an analytical review of these able lectures. The work certainly deserves to receive more than an ordinary and passing notice. We must, however, defer the publication of the analysis until our next number. We are much struck with the novelty of many of the views of this eminent divine and wit. We particularly direct our readers' attention to the chapter "On the Faculties of Animals as compared with those of Men," as it contains a fund of anecdote, original thought, and racy humour. In our next number, we hope to do the author full justice.

*Of the Nature, Causes, and Treatment of Palsy and Apoplexy, &c.* By JAMES COPLAND, M.D., F.R.S. London: Longman. 1850.

THE greater portion of this work has already been published, in the "Dictionary of Practical Medicine," edited by Dr. Copland, and some of the chapters formed the substance of the Croonian Lectures delivered at the Royal College of Physicians in the years 1846-7. Dr. Copland is too well known to the profession to need any commendation from us. His "Dictionary of Practical Medicine" constitutes one of the literary pyramids of the age in which we live, and speaks with "trumpet tongue" of the editor's extraordinary industry, sagacity, knowledge, and perseverance. The work before us being in part a re-publication of a portion of the Dictionary, necessarily partakes more of the character of a compilation than that of an original treatise. We certainly should have been better pleased if the author had given us more of his *own* views, and less of the opinions of others. Combined, however, with the literature of each point discussed, Dr. Copland frequently speaks in his own proper person, and then we immediately perceive the discerning, observing, and reflecting physician. It is this circumstance that makes us the more regret the frequent references to the diversified and conflicting opinions of others with which the work is so replete. However, with this exception, we offer the author our meed of praise, and congratulate the profession on the appearance of his work. It will, we have no doubt, soon find its way into the library of every reading man in the profession.

*Etherology and the Phreno-Philosophy of Mesmerism and Magic, Eloquence, &c.* By J. STANLEY GRIMES, revised and edited by W. G. LE DUC. Boston and Cambridge, United States. 1850.

WE think the less that is said of this work the better the author will be pleased. We could, if we were so disposed, deal some heavy critical blows, but we feel reluctant to say anything offensive to the feelings of the aspirant for fame who has allowed his name to be published on the title page of this volume. It is much to be regretted that so much paper, composition, and print should have been expended in *such a cause;* but paper-manufacturers, printers, bookbinders, to say nothing of critics, *must live*, and trunk-makers occasionally require the means of giving to their productions an internal tunic. Thus the world wags. Our author, if he has no other consolatory reflection, may comfort himself with the assurance of doing the "state some service," in thus contributing his mite to the support of a section of his fellow-men with much more humble pretensions than he appears to lay claim to.

*On Instinct and Reason.* By ALFRED SMEE, F.R.S. Reeve, Benham, and Reeve: London. 1850.

THIS volume contains a fund of interesting anecdote, having reference to the subject of instinct. We do not, however, agree with the author's generalizations; nevertheless, the work is likely to find readers and admirers. The volume contains some excellent illustrations. We particularly refer to some admirably coloured engravings of injected animal tissues. We cannot close this short notice without directing the author's attention to a curious and unexplained circumstance—How is it that the history of the "*Aphis Vastator*" is printed in the chapter on "Perverted Reason?" Surely our friend Mr. Smee is a bit of a wag.

*Researches on the Natural History of Death.* By BENNET DOWLER, M.D. New Orleans. 1850.

A VERY interesting and instructive essay, by a sensible physician. The subject of premature interment has occupied much attention on the other side of the channel. We are not astonished at this when we hear that the number of premature interments in France prevented in *one year* amounted to ninety-four! Of these, thirty-five persons awoke of themselves from their lethargy at the moment the funeral ceremony was about to take place; thirteen recovered in consequence of the affectionate care of their families; seven revived upon the coffin in which they were inclosed falling; nine owed their recovery to wounds inflicted by the needle in sewing their winding-sheet; three to the sensation of suffocation they experienced in their coffin; nineteen to their interment having been delayed by fortuitous circumstances; and six to their interment having been delayed in consequence of doubts being entertained of their death. Dr. Dowler refers to the extraordinary French story of a lady who died and was buried. An old lover hearing of the fact, undertook a long journey to France to procure a lock of her hair. He disinterred the body at night for this purpose, and was rejoiced to see his mistress alive. As the lady had the misfortune to be married, the lovers fled to America, and after the expiration of twenty years returned to France. The husband, Mr. Rennelle, claimed his wife. The question came before the judicial tribunals, and the court awarded the body to the party who disinterred her, and restored her to life. Dr. Dowler gravely expresses his disbelief in the truth of the narrative; and with great *naivete* asks, "How *could* the lady live so long underground without *fuel, food,* and *air?*" We would add—without the use of a *toothpick?* Dr. Dowler refers to the *penchant* which exists in France for resuscitated wives marrying previously rejected lovers, which he thinks may possibly explain a maxim ascribed to a French philosopher, viz.:—*That marry whom you will, you will afterwards find that you have married quite a different person.* Pleasant discovery!

*Memoir of Dr. Prichard.* By Dr. J. A. Symonds. Bristol. 1850. Pamphlet.

We have to apologise to the author of this pamphlet for not before directing the attention of our readers to his able, interesting, and classical piece of instructive medical biography. Dr. Symonds gives us a rapid but correct account of the career of the late Dr. Prichard, with an admirable résumé of his numerous writings. The pamphlet does great credit to the intellect and heart of the author. We regret that our space will not admit of our publishing a more detailed account of this memoir. All who are interested in the life of the distinguished ethnologist must procure the pamphlet. It will repay perusal.

*Lunacy and Lunatic Life.* By A Late Superintendent of an Asylum. London. 1850.

This volume contains a popular account of lunacy and lunatic asylums, and will be found of service to all who have friends and relatives mentally afflicted. Its pretensions are of a humble character; but, nevertheless, it may be consulted with advantage.

